# The Differences in Brain Activity between Narrow Band Noise and Pure Tone Tinnitus

**DOI:** 10.1371/journal.pone.0013618

**Published:** 2010-10-27

**Authors:** Sven Vanneste, Mark Plazier, Elsa van der Loo, Paul Van de Heyning, Dirk De Ridder

**Affiliations:** 1 Brai2n, Tinnitus Research Institute (TRI) and Department of Neurosurgery, University Hospital Antwerp, Edegem, Belgium; 2 Brai2n, Tinnitus Research Institute (TRI) and ENT, University Hospital Antwerp, Edegem, Belgium; University of Regensburg, Germany

## Abstract

**Background:**

Tinnitus is an auditory sensation characterized by the perception of sound or noise in the absence of any external sound source. Based on neurobiological research, it is generally accepted that most forms of tinnitus are attributable to maladaptive plasticity due to damage to auditory system. Changes have been observed in auditory structures such as the inferior colliculus, the thalamus and the auditory cortex as well as in non-auditory brain areas. However, the observed changes show great variability, hence lacking a conclusive picture. One of the reasons might be the selection of inhomogeneous groups in data analysis.

**Methodology:**

The aim of the present study was to delineate the differences between the neural networks involved in narrow band noise and pure tone tinnitus conducting LORETA based source analysis of resting state EEG.

**Conclusions:**

Results demonstrated that narrow band noise tinnitus patients differ from pure tone tinnitus patients in the lateral frontopolar (BA 10), PCC and the parahippocampal area for delta, beta and gamma frequency bands, respectively. The parahippocampal-PCC current density differences might be load dependent, as noise-like tinnitus constitutes multiple frequencies in contrast to pure tone tinnitus. The lateral frontopolar differences might be related to pitch specific memory retrieval.

## Introduction

Tinnitus is an auditory sensation characterized by the perception of sound or noise in the absence of any external sound source. Therefore it is also called an auditory phantom percept[1], similar to phantom pain [2], [3], and it is present in 10 to 15% of the population [4], [5]. Tinnitus can be extremely disruptive and debilitating leading many patients to seek medical attention. Based on neurobiological research, it is generally accepted that most forms of tinnitus are attributable to maladaptive plasticity due to damage to auditory system [6], [7]. Changes in the inferior colliculus, the thalamus and the auditory cortex have been demonstrated [8], [9], [10], [11], [12], [13]. Alterations of neural activity were also observed in non-auditory brain structures [14], [15], [16].

The heterogeneity of the results encountered in the above mentioned studies limit the understanding of the pathophysiology of tinnitus. The variability of the results suggests the existence of different tinnitus subgroups, which differ not only in their clinical characteristics (e.g. hearing loss or no, bilateral vs. unilateral tinnitus, pure tone vs. narrow band noise, tinnitus accompanied with distress or not, etc.). It has been shown that the amount of tinnitus suppression obtained depends on the tinnitus characteristics and stimulation design used, both for Transcranial Magnetic stimulation (TMS)[17], [18], [19], [20] and implanted electrodes[21]: pure tone tinnitus can be suppressed equipotentially by tonic and burst stimulation, whereas noise-like tinnitus can best be suppressed by burst TMS and burst electrical stimulation. This suggests that also the underlying neurophysiological mechanism of pure-tone and noise-like tinnitus might differ.

No study has yet investigated the neurophysiological differences in the characteristics of tinnitus sound perception (narrow band noise vs. pure tone) between tinnitus patients, although this could lead to a better understanding of pathological auditory neural activity. However, in the literature it was already hypothesized that pure tone tinnitus may be the result of increased tonic firing in the tonotopic lemniscal (classical) system, while narrow band tinnitus can be caused by increased burst firing in the non-tonotopic extralemniscal (non-classical) system [17], [18]. Furthermore, single cell recordings studies in rhesus monkeys showed that neurons in lateral belt areas of the auditory cortex were more specifically activated by complex sounds containing a broad range of frequencies than by pure tones [22], [23]. These authors also measured neural responses in the lateral belt areas elicited by band-passed noises differing in center frequency and bandwidth [24]. In addition, Blood et al. [25] demonstrated in a PET study that when subtracting brain images listening to noise from those while listening to tones, increased regional cerebral blood flow was found in the right prefrontal cortex (BA 10), and decreased regional cerebral blood flow in the precuneus and the right parahippocampal area.

The objective of the present study was to verify the neurophysiological differences between pure tone and narrow band noise in a homogenous but large group of tinnitus patients using source localized resting state EEG recordings. Quantitative analysis of EEG is a low-cost and useful neurophysiological approach to the study of physiology and pathology [26]. Cortical sources of the EEG rhythms were estimated by standardized low-resolution brain electromagnetic tomography (sLORETA)[27]. sLORETA is a functional imaging technique estimating maximally smoothed linear inverse solutions accounting for distributed EEG sources within MNI space [27]. This feature is of special importance for the comparison of EEG results with those of most structural and functional neuroimaging studies. sLORETA has been successfully used in recent EEG studies on tinnitus [28], [29].

## Results

### Power Spectra

**Figure 1:**
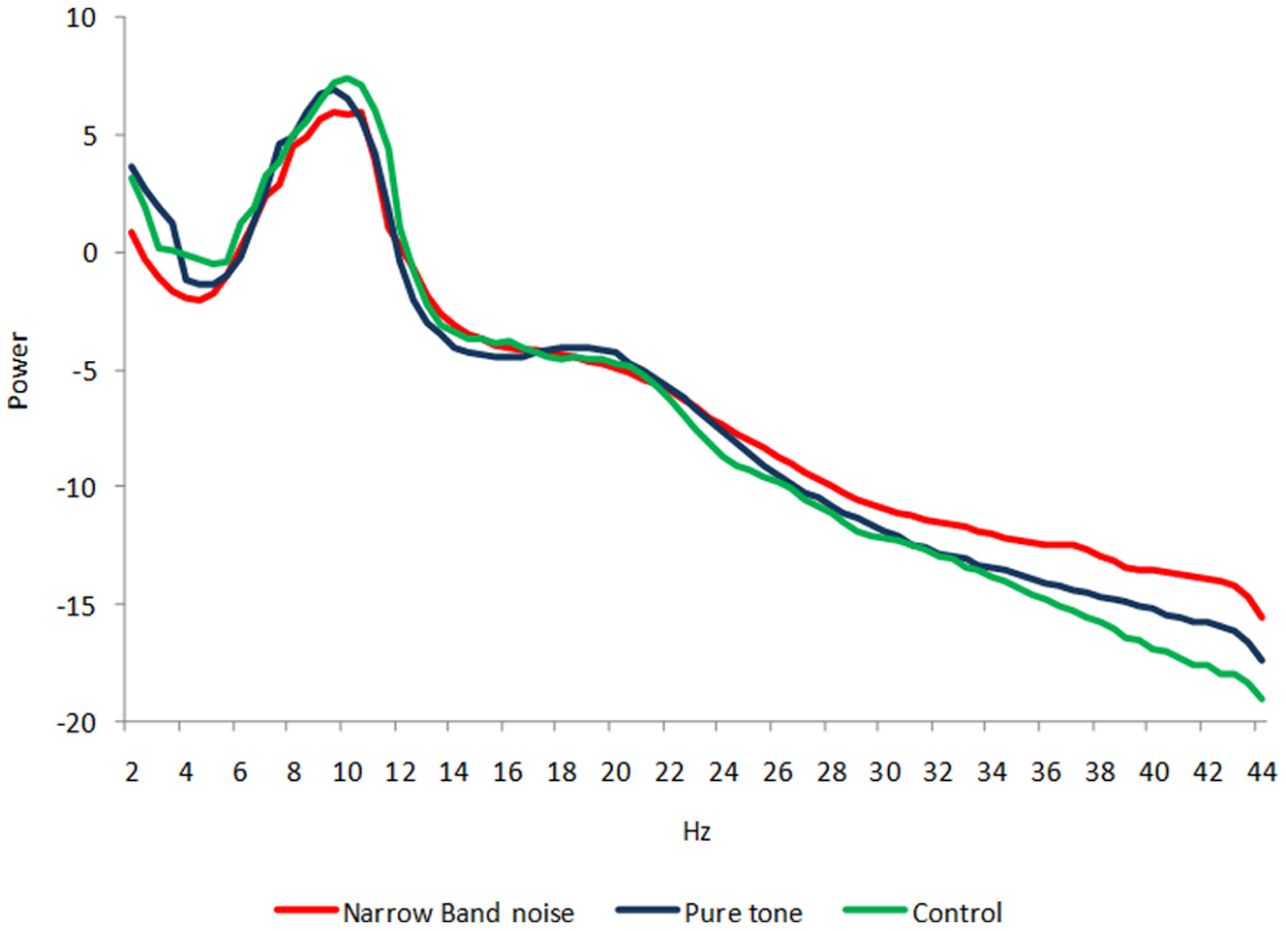
EEG power in tinnitus patients for narrow band noise tinnitus patients, pure tone tinnitus patients and control subjects, averaged over all electrodes.

**Figure 2:**
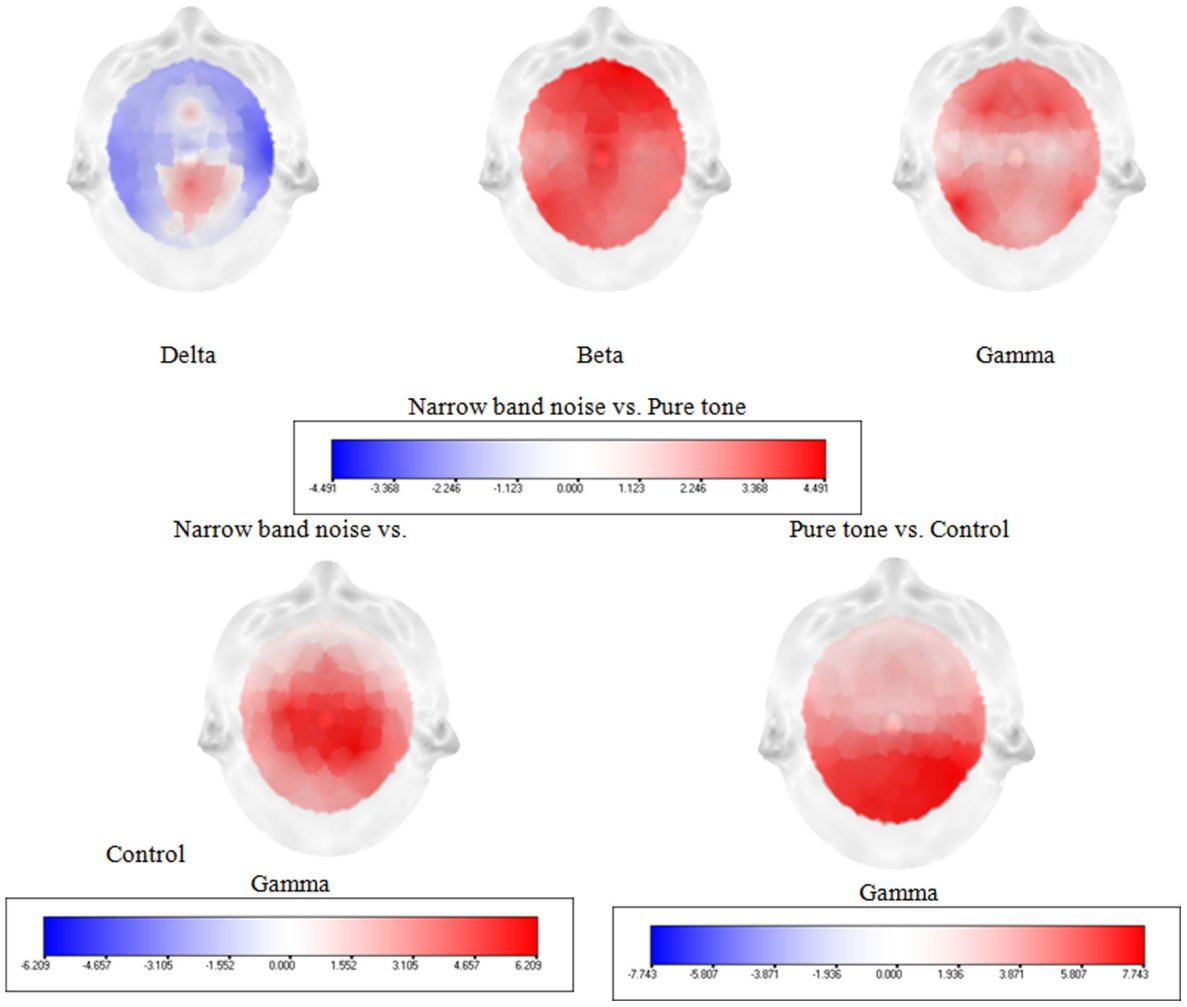
Electrode comparison of power spectra for narrow band noise tinnitus patients, pure tone tinnitus patients and control subjects.

The distribution across pure tone and narrow band noise groups was significantly higher (*p*<.05) in delta (2–3.5 Hz), beta (25–30 Hz) and gamma (30.5–44 Hz) frequency bands (see Fig 1). After establishing a significant difference between pure tone and narrow band noise in spectra averaged over all electrodes, it is of interest to know which electrodes contributed most to this difference and at which frequency band. Analysis performed for electrode and tinnitus type indicates a decrease in delta power for narrow band noise in comparison to pure tones in the right frontal-parietal region, and an increase in beta and gamma in centro-frontal and left occipital regions (see Fig 2; *p*<.05).

A similar analysis for pure tone and control subjects, and narrow band noise and control subjects yielded only significant differences for the gamma (30.5–44 Hz) frequency band (see Fig 1). Analysis performed for electrode and tinnitus type and control subjects indicates an increase in gamma power for narrow band noise in comparison to control subjects (*p*<.05) in centro-occipital regions and an increase in gamma power for pure tones in comparison to control subjects (*p*<.05) in centro-frontal and centro-occipital regions (see Fig 2).

### Neural Generators: Narrow-Band-Noise vs. Pure Tone

**Figure 3:**
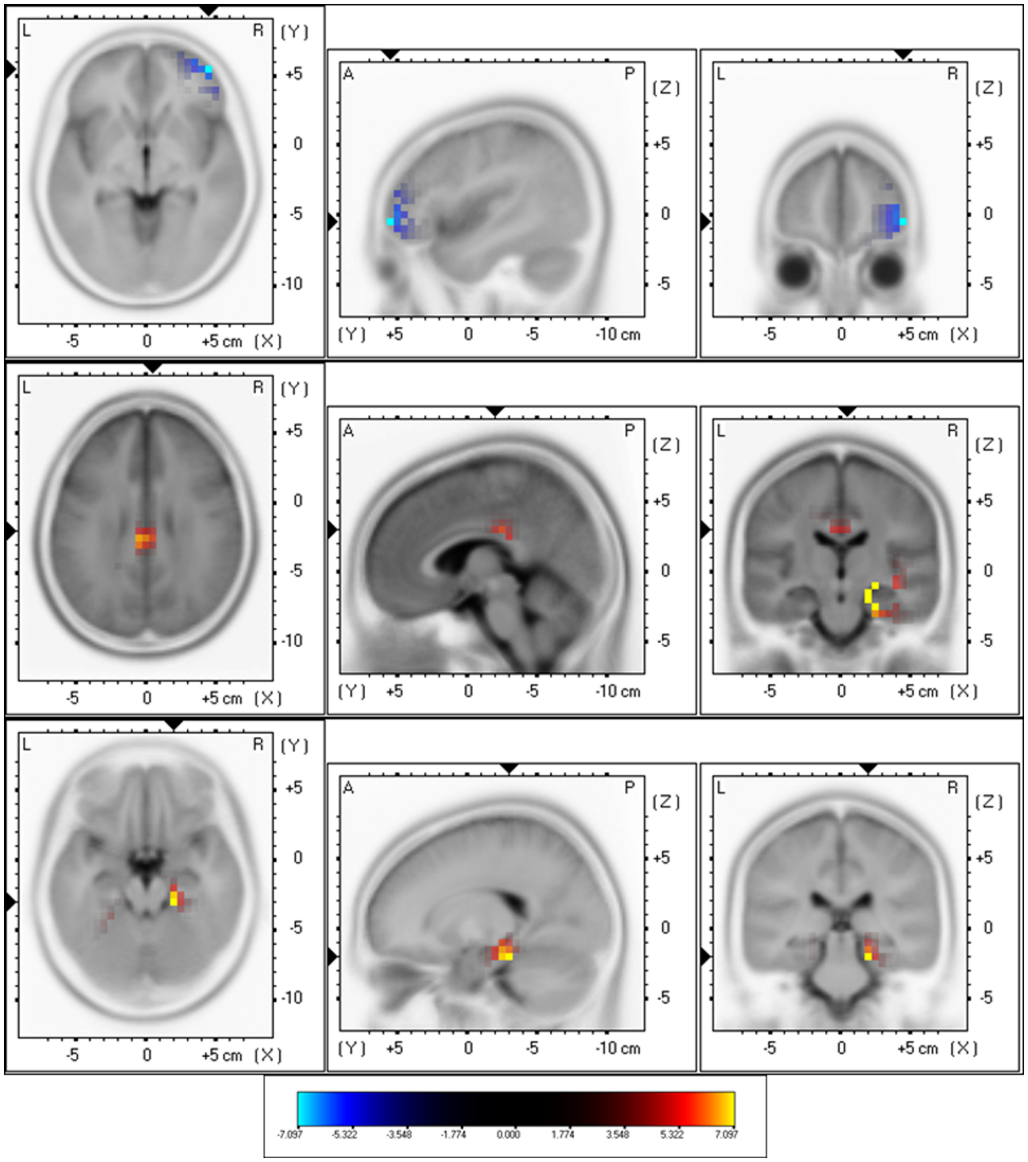
sLORETA contrast analysis between Narrow-Band-Noise versus Pure Tone tinnitus (*p*<.05). Decreased activity within Delta (1–3.5 Hz; Top Panel) in the prefrontal cortex (BA10), and increased activity within Beta (25–30 Hz; Middle Panel) and Gamma (30.5–45 Hz; Bottom Panel) in respectively posterior cingulate cortex (PCC; BA23) and the right parahippocampal area (BA35) for patients presenting with bilateral narrow band tinnitus in comparison to bilateral pure tone tinnitus.

The sLORETA showed significant differences between Narrow-Band-Noise and Pure Tone tinnitus patients. Decreased synchronized delta activity could be revealed in the right lateral frontopolar cortex (BA10) for narrow-band noise patients in comparison to pure tone patients. Increased synchronized beta could be found in the posterior cingulated cortex (PCC; BA23) and the right hippocampal area (BA35) (see Fig 3; *p*<.05) for narrow-band noise patients in comparison to pure tone patients. Also increased gamma activity was found in the right parahippocampal area (BA35)(see Fig 3; *p*<.05) for narrow-band noise patients in comparison to pure tone patients.

### Narrow-Band-Noise vs. Control subjects

**Figure 4:**
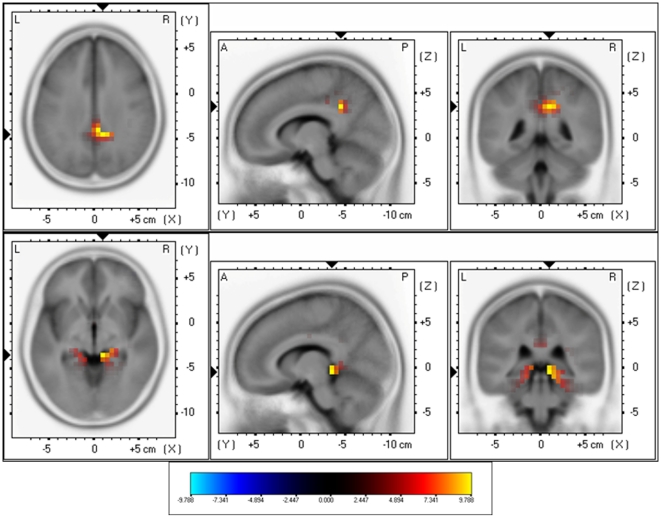
sLORETA contrast analysis between Narrow-Band-Noise tinnitus patients versus Control subjects (*p*<.05). Increased activity within Beta (25–30 Hz; Top Panel) in the posterior cingulate cortex (PCC; BA31) and Gamma (30.5–45 Hz; Bottom Panel) in the right and left parahippocampal areas (BA35) for bilateral narrow band tinnitus in comparison to controls.

Furthermore, sLORETA demonstrated significant differences between Narrow-Band-Noise tinnitus patients and the control subjects. Increased synchronized beta (25–30 Hz) in the PCC (BA31) and gamma (40.5–45 Hz) activity could be found and the left parahippocampal area (BA35) respectively (see Fig 4; *p*<.05) for narrow-band noise patients. No significant differences could be retrieved in the delta frequency.

### Pure Tone vs. Control subjects

**Figure 5:**
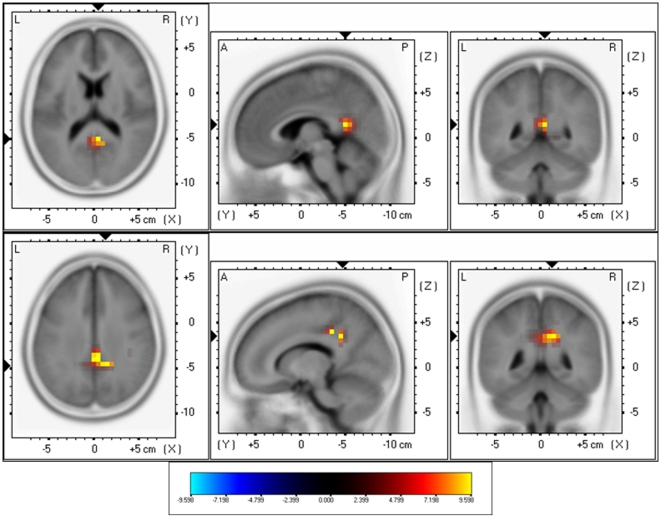
sLORETA contrast analysis between Pure Tone tinnitus patients versus Control subjects (*p*<.05). Increased activity within Beta (25–30 Hz; Top Panel) and Gamma (30.5–45 Hz; Bottom Panel) in the posterior cingulate cortex (PCC; BA31) for patients with bilateral pure tone tinnitus in comparison to controls.

When comparing Pure Tone tinnitus patients with the Controls subjects, significant differences were revealed for beta (25–30 Hz) and gamma (40.5–45 Hz) activity. For both increased synchronized activity could be found in the PCC (BA31) for pure tone tinnitus patients (see Fig 5; *p*<.05). No significant differences could be retrieved in the delta frequency range.

### Region of interest analysis

A significant effect was found for the log-transformed current density for the different groups on the region of interest for the gamma frequency band, *F*(8,239) = 3.946, *p*<.001 (see Table 1). Univariate ANOVA further yielded a significant effect for left primary auditory cortex (*F*(2, 119) = 12.98, *p*<.001), right primary auditory cortex (*F*(2, 119) = 9.84, *p*<.001), left secondary auditory cortex (*F*(2, 119) = 8.62, *p*<.001) and right secondary auditory cortex (*F*(2, 119) = 5.35, *p*<.01) respectively. A Bonferroni multiple comparison analysis (*p*<.05) revealed that the control subjects had significant lower log averaged current density in comparison to pure tone and narrow band noise tinnitus patients. Pure tone and narrow band noise tinnitus patient did not differ from each other. No significant effect was obtained for delta (*p*>.56) and beta (*p*>25).

**Table 1:** Region of interest analyses for gamma band frequency (log-transformed current density).

		Pure Tone	Narrow Band noise	Control
primary AC	left	1.42^a^	1.65^a^	0.99^b^
	right	1.45^a^	1.57^ a^	1.02^ b^
secundary AC	left	1.89^a^	2.12^ a^	1.55^ b^
	right	1.91^a^	2.03^ a^	1.51^ b^

Statistical analyses (ANOVA) comparing the differences between the mean scores for the respective groups. Subscript show the significant differences (*p*<.05) between pure tone, narrow band noise and control subjects (Bonferroni correction for multiple comparisons). That is, numbers with a different subscript differ significantly from each other. In general control subjects have a lower current density in the left and right primary and secondary auditory cortex in comparison to pure tone and narrow bands noise tinnitus patients.

### Hearing loss

No significant difference was found for hearing loss between Narrow-Band-Noise and Pure Tone (*p* = .71), as measured by the loss in decibels (dB SPL) at the tinnitus frequency.

## Discussion

The aim of the present study was to detect neurophysiological differences in narrow band noise compared to pure tone tinnitus to get further insights into the pathophysiology of phantom sound perception in tinnitus patients. This study shows global power changes of the spontaneous EEG activity. A general decrease in the delta frequency range and a general increase in the beta and gamma frequency range were found when comparing narrow band noise with pure tone tinnitus and an increase in gamma frequency range when comparing narrow band and pure tone with control subjects respectively. These power changes were further confirmed on individual electrode analysis. This study demonstrated that narrow band noise tinnitus patients differ from pure tone tinnitus patients in the lateral frontopolar cortex (BA 10), PCC and the parahippocampal area for the delta, beta and gamma frequency bands, respectively. Furthermore, a comparison between narrow band noise and a normative database revealed increased synchronized activity in the PCC and the beta and in the parahippocampal area for gamma. Also pure tone tinnitus is characterized by increased synchronized activity for beta and gamma in the PCC in comparison to a normative database.

Our results revealed differences in delta, beta and gamma activity when comparing narrow band noise with pure tone tinnitus and beta and gamma activity when comparing pure tone tinnitus and narrow band noise tinnitus with control subjects.

Although spectral analysis only showed gamma differences, source analyzed current density also demonstrated focal beta activity in the deep seated PCC. As the spectral analysis calculated the average over all electrodes, this could lead to a loss of sensitivity for small focalized activity with low power, characteristic for high frequency activity.

Pure tone presentation activates the frontopolar cortex [30], and the right prefrontal cortex is involved in memory for pitch for pure tones [31], as well as for the on-line maintenance and encoding of tonal patterns [32]. The delta activity differences in the right lateral frontopolar cortex (BA 10) between noise-like tinnitus and pure tone tinnitus might therefore reflect the fact that in noise-like tinnitus no specific memory for pitch is evoked in contrast to pure tone tinnitus.

The posterior auditory cortex is anatomically connected to the posterior cingulate and parahippocampal area [33], [34]. Auditory input to the PCC is directed to area 23, and not to areas 29 and 30 [35]. This is in accordance with the PCC beta and gamma band activity noted in BA31 in both pure tone and noise-like tinnitus. The PCC has been associated with cognitive evaluation and memorization of sensory input [36].

The posterior parahippocampal area is the main node of entry for auditory information to the medial temporal lobe memory system, where salient information is encoded into long-term memory [37]. It has therefore been associated with learning and memory processing [38], [39], [40]. The posterior parahippocampal area is furthermore involved in sensory gating of incoming irrelevant or redundant auditory input [41].

Involuntary conscious memory recall in comparison to voluntary memory recall is related to activation of the PCC (extending to the precuneus) and parahippocampal area [42]. Therefore it could be hypothesized that the combined PCC-parahippocampal activation might be related to constant involuntary recall of a phantom sound from auditory memory. There might be more activation in noise-like tinnitus than in pure tones in these two areas because noise-like tinnitus contains a spectrum of frequencies whereas pure tones contain just a one or a limited amount of frequencies. The current density differences might therefore reflect load dependent differences.

The sLORETA analysis did not yield differences in the auditory cortex. However, region of interest analyses revealed significant differences in the left and right primary and secondary auditory cortex for the gamma frequency for tinnitus patients in comparison to control subjects. Previous research already demonstrated that tinnitus is correlated to sustained high frequency gamma band activity in temporal areas in humans in QEEG [43] and MEG studies [44], [45].

It might seem as a surprise that no differences were found in the auditory cortex for narrow band noise vs. pure tone tinnitus. Previous research using single cell recordings studies in rhesus monkeys showed that neurons in lateral belt areas of the auditory cortex were more specifically activated by complex sounds containing a broad range of frequencies than by pure tone [22], [23]. These authors also measured neural responses in the lateral belt areas elicited by band-passed noises differing in center frequency and bandwidth [24]. One possibility might be that the auditory cortex only codes for tinnitus intensity discrimination [46], and since both groups are characterized by the same VAS intensity scores, subtracting the two groups results in an absence of activation. Yet, another methodological issue might be involved. Because we used sLORETA - which is known to have a low-resolution brain tomography - it is possible that higher resolution techniques might find differences in the auditory cortex between narrow band noise and pure tone tinnitus patients, analogous to what has been found in single cell recordings.

In summary, we conclude that differences in brain activity can be found in the parahippocampal area, the PCC and the frontopolar cortex (BA 10) between narrow band noise and pure tone tinnitus. The PCC and parahippocampal differences could be load dependent whereas the lateral frontopolar differences might be related to pitch specific memory retrieval.

## Materials and Methods

### Participants

Eighty-two patients with bilateral tinnitus (N = 82; 51 males and 31 females) with a mean age of 49.35 (range: 17–81 years) were selected from the multidisciplinary Tinnitus Research Initiative (TRI) Clinic of the University Hospital of Antwerp, Belgium. Individuals with pulsatile tinnitus, Ménière disease, otosclerosis, chronic headache, neurological disorders such as brain tumors, and individuals being treated for mental disorders were not included in the study in order to obtain a homogeneous sample. Fifty-three patients presented with narrow-band noise tinnitus, while twenty-nine with pure tune tinnitus. No significant differences (unpaired *t*-test) were found between narrow band noise and pure tone tinnitus patients for tinnitus duration, VAS intensity, VAS distress, HADS depression and HADS anxiety (see Table 2).

**Table 2:** Mean and standard deviation scores on tinnitus duration, VAS intensity, VAS distress, HADS anxiety and HADS depression for pure tone and narrow band noise tinnitus patients.

	Pure Tone	Narrow Band Noise	*p-*value
Gender	♂ 65.52%, ♀ 34.48%	♂ 62.26%, ♀ 37.74%	
Tinnitus Duration (years)	6.41 (6.54)	6.00 (6.72)	.75
VAS Intensity	5.80 (2.18)	6.58 (2.35)	.18
VAS Distress	5.78 (2.09)	6.38 (2.61)	.16
HADS Anxiety	7.10 (3.27)	6.76 (2.83)	.69
HADS DepressionHearing loss	7.52 (3.67)13.60 (16.25)	8.36 (3.65)16.81 (17.44)	.40.45

HADS =  Hospital Anxiety Depression Scale.

All patients were investigated for the extent of hearing loss using audiograms. Tinnitus matching was performed looking for tinnitus pitch (frequency) and tinnitus intensity. Participants were requested to refrain from alcohol consumption 24 hours prior to recording, and from caffeinated beverages consumption on the day of recording.

This study was approved by the local ethical committee (Antwerp University Hospital) and was in accordance with the declaration of Helsinki.

### EEG data collection

EEGs were obtained in a fully lighted room with each participant sitting upright in a comfortable chair. The EEG was sampled with 19 electrodes (Fp1, Fp2, F7, F3, Fz, F4, F8, T7, C3, Cz, C4, T8, P7, P3, Pz, P4, P8, O1 O2) in the standard 10–20 International placement referenced to linked ears and impedances were checked to remain below 5 kΩ. Data were collected for 100 2-s epochs eyes closed, sampling rate  = 1024 Hz, and band passed 0.15–200 Hz. Data were resampled to 128 Hz, band-pass filtered (fast Fourier transform filter) to 2–44 Hz. These data were transposed into Eureka! Software [47]), plotted and carefully inspected for manual and ICA dependent artifact-rejection. All episodic artifacts including eye blinks, eye movements, teeth clenching, body movement, or ECG artifacts were removed from the stream of the EEG.

### Power spectral analyses

A digital FFT-based power spectrum analysis (Time Domain Tapering: Hamming, Frequency Domain Smoothing: Blackman, Overlapping FFT Windows Advancement Factor: 8) computed the power density of EEG rhythms with 0.5 Hz frequency resolution.

In order to summarize the data and because spectra from all electrodes demonstrated similar shape and scale, we averaged the log transformed spectra of all 19 scalp electrodes for each subject. We then averaged these individual spectra to one spectrum for the pure tone and narrow band noise tinnitus groups.

For group wise comparison, the power spectra of both groups were compared with independent samples *t-*test for each frequency point; no correction for multiple comparisons was applied similar to Moazami-Goudarzi et al. [28] and therefore considered as exploratory for frequency point comparison.

For band power comparison the methodology used is non-parametric. It is based on estimating, via randomization, the empirical probability distribution for the max-statistic (e.g. the maximum of a *t*), under the null hypothesis. This methodology corrects for multiple testing (i.e., for the collection of tests performed for all electrodes, and for frequencies). Due to the non-parametric nature of the method, its validity does not have to rely on any assumption regarding normal distributions. Complete overview of the methodology, with details about the properties (e.g. pertaining to its non-parametric nature, and pertaining to how it properly corrects for multiple testing) can be found in Nichols and Holmes [48].

### Control subjects

Similar to the tinnitus patients, EEGs (Mitsar, Nova Tech EEG, Inc, Mesa) were obtained for a control group (N = 40; 27 males and 13 females) in a fully lighted room with each participants sitting upright in a comfortable chair. None of these subjects were known to suffer from tinnitus or hearing loss. Exclusion criteria for the control subjects were known psychiatric or neurological illness, drug/alcohol abuse, current psychotropic/CNS active medications, history of head injury (with loss of consciousness) or seizures. The mean age of the control subjects was 49.29 years (range: 17–75 years). The control group was matched for age and gender. The EEG was sampled with 19 electrodes (Fp1, Fp2, F7, F3, Fz, F4, F8, T7, C3, Cz, C4, T8, P7, P3, Pz, P4, P8, O1 O2) in the standard 10–20 International placement referenced to linked lobes and impedances were checked to remain below 5 kΩ. Data were collected for 100 2-s epochs eyes closed, sampling rate  = 1024 Hz, and band passed 0.15–200 Hz. Data were resampled to 128 Hz, band-pass filtered (fast Fourier transform filter) to 2–44 Hz. The data were cleaned-up in a similar way to the tinnitus patients by manual artifact rejection and ICA. Again to investigate the effect possible ICA component rejection we compared the power spectra in two approaches: (1) after visual artifact rejection only (before ICA) and (2) after additional ICA component rejection (after ICA). To test for significant differences between the two approaches we performed a repeated-measure ANOVA, considering mean band power as within-subject variables.

### Source Localization

sLORETA was used to estimate the intracerebral electrical sources that generated the scalp-recorded activity in each of the eight frequency bands [27]. sLORETA computes electric neuronal activity as current density (A/m^3^) without assuming a predefined number of active sources (blind source separation). The sLORETA solution space consists of 6,239 voxels (voxel size: 5×5×5 mm) and is restricted to cortical gray matter and hippocampi, as defined by digitized MNI152 template [49]. Scalp electrode coordinates on the MNI brain are derived from the international 10/5 system [50]. sLORETA has received considerable validation from studies combining LORETA with other more established localization methods, such as functional Magnetic Resonance Imaging (fMRI) [51], [52], structural MRI [53], Positron Emission Tomography (PET) [54], [55], [56]. Further sLORETA validation has been based on accepting as ground truth the localization findings obtained from invasive, implanted depth electrodes, in which case there are several studies in epilepsy [57], [58] and cognitive ERPs [59]. It is worth emphasizing that deep structures such as the anterior cingulate cortex [60], and mesial temporal lobes [61] can be correctly localized with these methods.

### sLORETA statistical analyses

In order to identify potential differences in brain electrical activity between conditions, sLORETA was then used to perform voxel-by-voxel between-condition comparisons of the current density distribution. Nonparametric statistical analyses of functional sLORETA images (statistical non-parametric mapping; SnPM) were performed for each contrast employing a t-statistic for unpaired groups and a corrected (P<0.05). As explained by Nichols and Holmes, the SnPM methodology does not require any assumption of Gaussianity and corrects for all multiple comparisons [48]. We performed one voxel-by-voxel test (comprising 6,239 voxels each) for the different frequency bands.

### Region of interest analysis

The log-transformed electric current density was averaged across all voxels belonging to the region of interest. Regions of interest were respectively the left and right primary auditory cortex (BA40 and BA41) and the left and right secondary auditory cortex (BA21 and BA22). Region of interest analyses were computed for the different frequency bands separately.

A multivariate ANOVA (i.e. Wilks' Lambda) for the frequency bands was used with the respective region of interest (i.e. left and right primary auditory cortex (BA40 and BA41) and left and right secondary auditory cortex (BA21 and BA22) as dependent variables and different groups (pure tone, narrow band noise and control subjects) as independent variable. A Bonferroni correction was applied for multiple comparisons.
